# Predicting mutations deleterious to function in beta-lactamase TEM1 using MM-GBSA

**DOI:** 10.1371/journal.pone.0214015

**Published:** 2019-03-19

**Authors:** Christopher Negron, David A. Pearlman, Guillermo del Angel

**Affiliations:** 1 Schrödinger, New York, New York, United States of America; 2 Alexion Pharmaceuticals Inc., Boston, Massachusetts, United States of America; Wake Forest University, UNITED STATES

## Abstract

Missense mutations can have disastrous effects on the function of a protein. And as a result, they have been implicated in numerous diseases. However, the majority of missense variants only have a nominal impact on protein function. Thus, the ability to distinguish these two classes of missense mutations would greatly aid drug discovery efforts in target identification and validation as well as medical diagnosis. Monitoring the co-occurrence of a given missense mutation and a disease phenotype provides a pathway for classifying functionally disrupting missense mutations. But, the occurrence of a specific missense variant is often extremely rare making statistical links challenging to infer. In this study, we benchmark a physics-based approach for predicting changes in stability, MM-GBSA, and apply it to classifying mutations as functionally disrupting. A large and diverse dataset of 990 residue mutations in beta-lactamase TEM1 is used to assess performance as it is rich in both functionally disrupting mutations and functionally neutral/beneficial mutations. On this dataset, we compare the performance of MM-GBSA to alternative strategies for predicting functionally disrupting mutations. We observe that the MM-GBSA method obtains an area under the curve (AUC) of 0.75 on the entire dataset, outperforming all other predictors tested. More importantly, MM-GBSA’s performance is robust to various divisions of the dataset, speaking to the generality of the approach. Though there is one notable exception: Mutations on the surface of the protein are the mutations that are the most difficult to classify as functionally disrupting for all methods tested. This is likely due to the many mechanisms available to surface mutations to disrupt function, and thus provides a direction of focus for future studies.

## Introduction

DNA sequencing has seen tremendous advances since the human genome was first sequenced in 2001 [1,2]. DNA sequencing cost is a great indicator of this progress as it has decreased by six orders of magnitude since the first sequenced human genome [3]. This in turn has facilitated the generation of large datasets of genetic variation [4–6]. Of particular note are the ExAC and gnomAD databases, which contain the full human exome (protein-coding region) for over 120,000 individuals [4]. However, despite these large collections of genetic variants, predicting the functional effect of a genetic variant remains a significant challenge for missense mutations [7]. Missense mutations refer to a single-amino acid change to a gene.

Missense variants play a critical role in medical diagnosis and discovery since certain missense variants have been strongly correlated with disease susceptibility in several human cancers [8,9]. The most noteworthy examples are missense variants in tumor suppressor genes BRCA1 and BRCA2 [8,10]. However, the challenge of utilizing missense variants for diagnosis is that many missense variants are not deleterious to function, appearing in the human exome at 7.9% of sites [4]. Additionally, the rarity of any one specific missense variant makes drawing a statistical link between function and missense variant challenging [11,12]. Furthermore, rare diseases contain another barrier to obtaining significant statistical links due to the rare occurrence of the disease itself. Lastly, further complicating the matter is the fact that experimental assays to assess how a specific missense variant impacts protein function only covers a limited space [8]—thereby further complicating experimental validation.

Several algorithms have been developed for predicting the functional outcome of a missense variant [7,13–15]. Many of these algorithms rely on generating a multiple sequence alignment (MSA) using the protein of interest, along with closely related sequences, typically from other species. The fundamental premise of this analysis is that conserved residues in the MSA are less tolerant to mutation. Some of the more robust tools in this space also incorporate structural information such as accessible surface area, B-factor, and hydrophobic propensity. Despite some success, these tools have shown significant sensitivity to the input sequence alignment, and thus performance varies across different systems [16].

In this work, we seek to benchmark a purely physics-based approach that is independent of sequence alignment known as MM-GBSA [17,18]. MM-GBSA predicts the relative change in folding free energy of an amino acid mutation using an implicit solvent model combined with a molecular mechanics energy function. MM-GBSA indirectly captures mutations that disrupt function by detecting mutations that significantly disrupt the stability of a protein. The link between protein stability and function has long been known and explored [19]. A central postulate arising from this field of analysis has been that if a protein is significantly destabilized, this will result in the protein misfolding, and consequently the mutation will disrupt protein function.

## Results

### Beta lactamase dataset

Datasets play an important role in any benchmark study. They need to be large enough to get meaningful statistics, and diverse enough to avoid bias in the data. Thus, we turned to a large scale mutational study of beta lactamase TEM-1 [20]. Beta lactamase TEM-1 is a bacterial enzyme responsible for hydrolyzing beta-lactam bonds that occur in beta-lactam antibiotics such as penicillin or amoxicillin. TEM-1 is well characterized both structurally and biochemically. And most importantly, the aforementioned study provides hundreds of examples of mutations that are both deleterious and neutral/beneficial to function. This in turn helps to mitigate bias in our study, and is the primary reason why we selected this system over human benchmarks, which lack substantial information on functionally neutral/beneficial mutations.

The beta lactamase TEM-1 dataset used in this study consists of 990 single-point mutations. Details of the functional assay performed on beta lactamase TEM-1 can be found in Jacquier *et al]*. Briefly, for each of the 990 mutations, cell growth was monitored at several concentrations of amoxicillin and the minimum concentration of amoxicillin required to inhibit cell growth was recorded, herein referred to as the minimum inhibitory concentration (MIC). The MIC measurement was converted to a MIC score via the following equation log_2_(MIC/500). 500 mg/L refers to the MIC measurement of the wildtype sequence. As a result, a MIC score of 0 would represent activity on par with the wildtype enzyme. MIC scores less than 0 are classified as deleterious in this paper. And MIC scores of 0 or greater are classified as neutral/beneficial.

**Fig 1:**
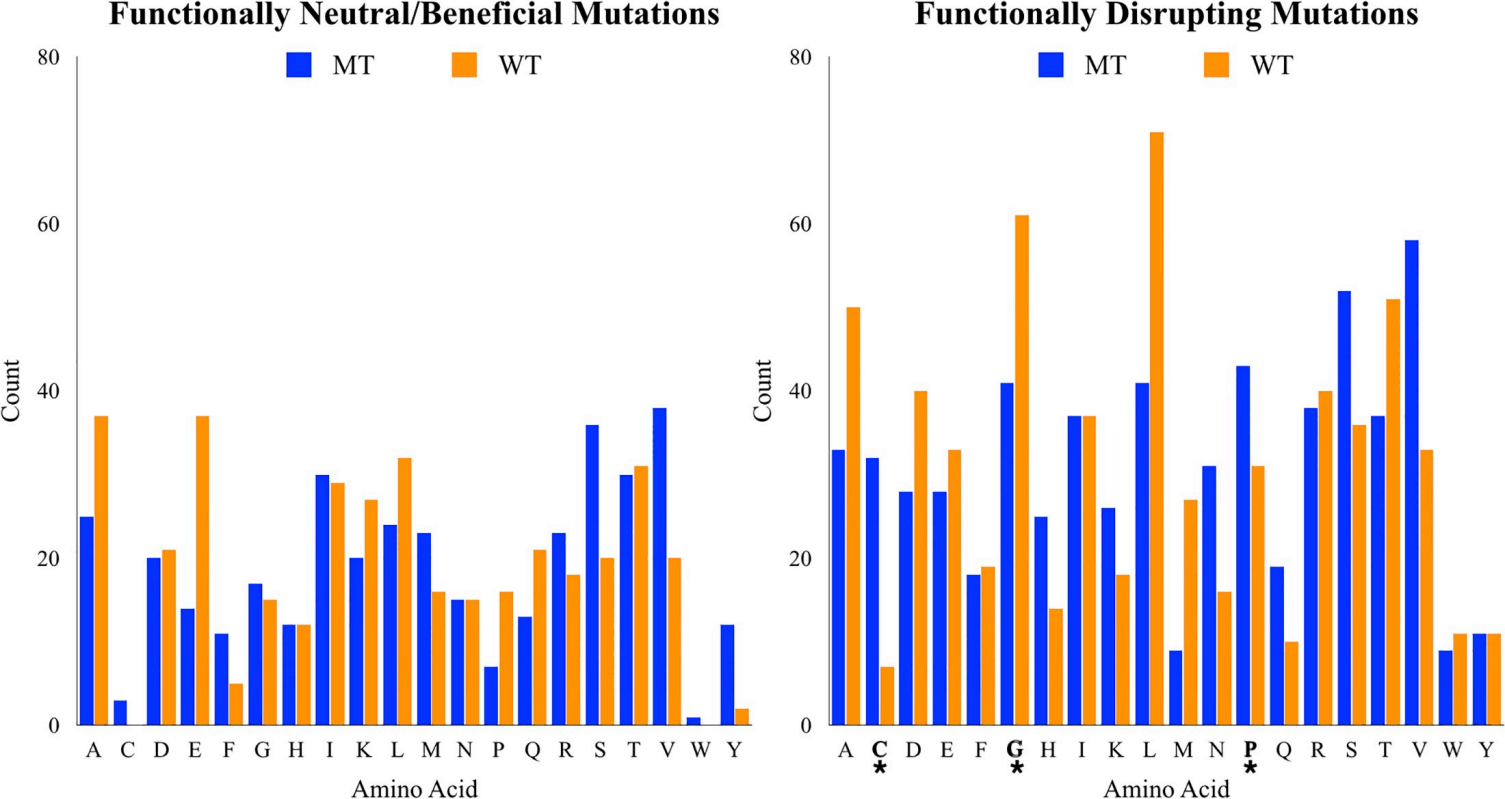
Frequency of appearance of amino acids in beta-lactamase dataset. The amino acid distribution is plotted for the functionally neutral/beneficial mutations (left) and the functionally disrupting mutations (right). The blue bars in the plot show the number of mutations from that wildtype amino acid. And the orange bars show the number of mutations to that amino acid. Functionally disrupting mutations that occur a statistical significant number of times (*p* < 0.01) are show in bold, with an asterisk under the amino acid.

The amino acid distribution of the functionally deleterious mutations and functionally neutral/beneficial mutations are shown in Fig 1. One benefit of this binary classification scheme is that it allows us to compare the observed frequency of each amino acid in the deleterious set to the mutation probabilities that would be expected by random selection from a binomial distribution. This makes it easy to identify mutation types that are significantly enriched in the functionally disrupting set.

Interestingly, three types of mutations appeared significantly (*p* < 0.01) more in the deleterious set than expected by random chance (Fig 1). These are mutations to cysteine, glycine, and proline, with cysteine being the most prominent enriched. Cysteine appears in the deleterious set 32 times while only appearing in the neutral/beneficial set 3 times. That is greater than a 10-fold enrichment in the deleterious set. Enrichment of cysteines in the deleterious set is likely tied to cysteine’s ability to participate in disulfide bonds that prevent proper protein folding. Prolines appear ~6.1 times more frequently in the deleterious set than in the neutral/beneficial set. The enrichment of proline in the deleterious set may be tied to the fact that proline occupies a unique dihedral space among natural occurring amino acids which can result in a strained geometry when the proline mutation is inserted into a non-proline position. Lastly, mutations to glycine are observed 2.4 times more frequently in the deleterious set versus the neutral/beneficial set. The small volume of the glycine side chain (a single hydrogen atom) likely creates large cavities in the core of a protein that in turn significantly destabilize the overall protein fold.

### Evaluating models

Looking beyond the statistical trends in the data, we sought to assess the performance of different models at classifying functionally deleterious amino acid mutations. To evaluate the models, we calculated a receiver operating characteristic (ROC) curve for each method. The area under the curve (AUC) is then measured to determine how well model separate deleterious mutations from the neutral/beneficial mutations. An AUC of 0.5 would represent a model that randomly classifies mutations as deleterious. An AUC of 1.0 would represent a model that perfectly agrees with the classification assigned by experiment.

**Fig 2:**
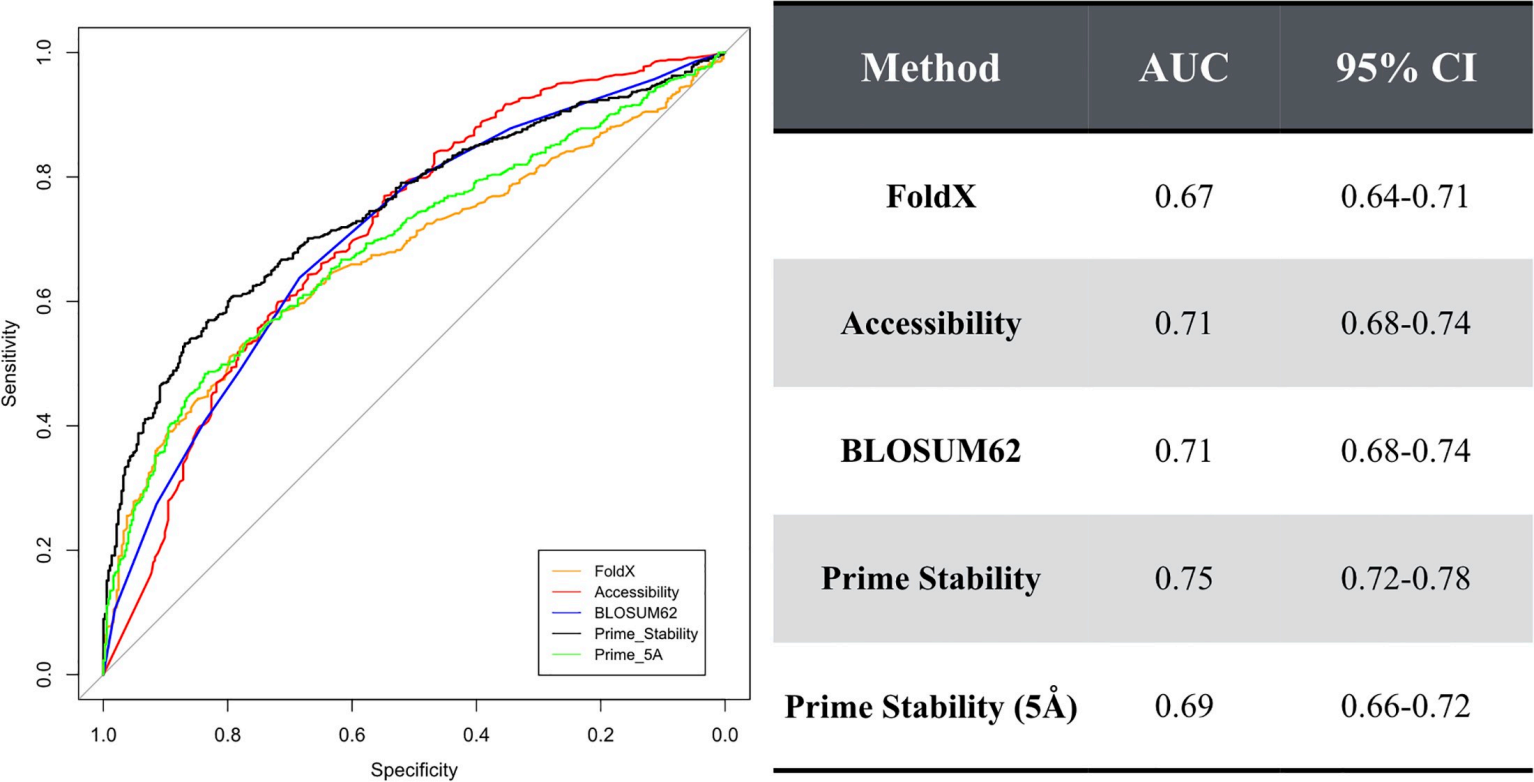
ROC curves for classifying functionally disrupting mutations on the beta lactamase dataset. This dataset is composed of 990 single-point mutations with measured changes in their minimum inhibitory concentration (MIC). The five models include energy scores from FoldX, the solvent accessibility of a side chain, the score from the BLOSUM62 substitution matrix, the Prime Stability score, and the Prime stability score with a 5Å minimization cutoff. The area under the curve (AUC) for each ROC curve is shown on the right along with its 95% confidence interval (CI).

Using AUC, we have benchmarked 5 different approaches for predicting mutations that are deleterious to function (Fig 2). The first approach is FoldX. FoldX is an empirical force field developed to predict changes in protein stability, and has been adapted for use in protein design [21,22]. FoldX obtains an AUC of 0.67 on this dataset, which is the lowest AUC among all the methods tested. Interestingly, a simple method such as the BLOSUM62 substitution matrix, a tool designed for generating sequence alignments, outperforms FoldX, obtaining an AUC of 0.71. This method works by predicting that the substitution of amino acids rarely observed in related protein families will result in a deleterious mutation. Another simple method, solvent accessibility, heretofore referred to as accessibility, also performs well on this dataset, with an AUC of 0.71. This method works by predicting that mutations at buried positions are more prone to be deleterious than mutations at solvent exposed positions. Lastly, we have benchmarked Schrodinger’s MM-GB/SA tool for predicting relative changes in protein stability, Prime [18]. Similar to FoldX, Prime predicts that deleterious mutations occur when significant increases in stability occur. Prime obtains the largest AUC, obtaining an AUC of 0.75. Lastly, we have benchmarked a variant of Prime that allowed greater side chain flexibility of neighboring residues by refining the side-chain of residues within 5 Å from the mutated side chain. Unexpectedly, this degrades Prime’s performance, obtaining an AUC of only 0.69.

**Fig 3:**
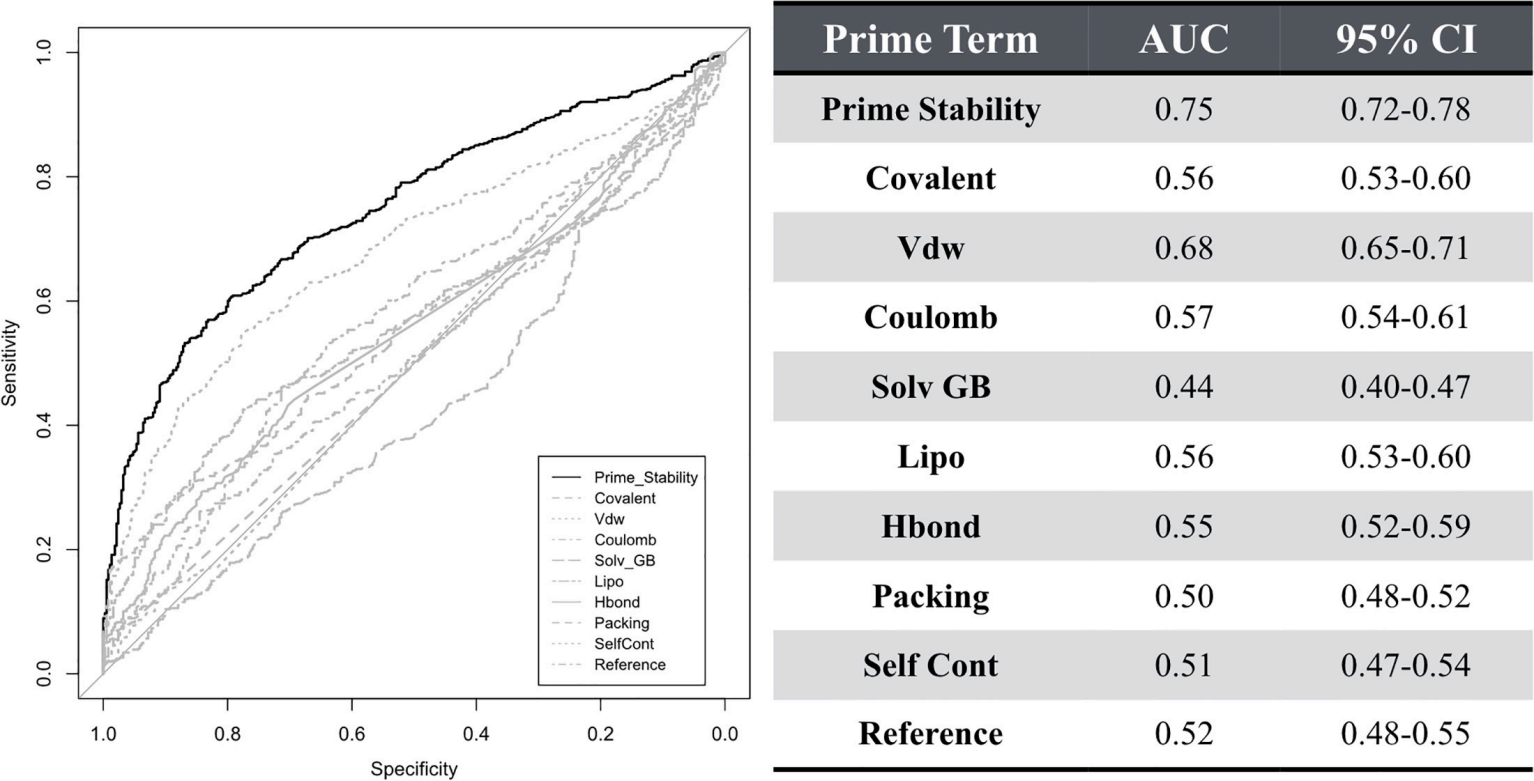
ROC curves for individual Prime terms on the beta lactamase dataset. The ROC curve for the Prime Stability energy function (black) and individual terms of the Prime Stability energy function (gray). The table on the right list the corresponding area under the curve (AUC) values and the 95% confidence intervals for the AUC values.

Additionally, we have examined how well each of the terms in the Prime Stability energy function perform individually. There are nine terms that are summed to calculate the Prime Stability score. The first term captures the energetics of covalent interactions (Covalent), such as bond angles and bond stretching. The second term models van der Waals interactions (VDW), which captures induced dipole interactions between atoms. The third term is a coulombic term (Coulomb), measuring electrostatic interactions. The fourth term is the generalized born term (Solv GB) which models the solvation and desolvation effects of amino acids. The fifth term is a measure of hydrophobic interactions with water, which is referred to as the Lipo term. The sixth term is a hydrogen bond term (Hbond). The seventh term is referred to as Packing and measures the quality of π-π interactions. The eighth term is a measure of self-interaction (Self Cont) of a side chain. This term captures side-chain hydrogen bonds with backbone atoms. Residues such as asparagine, glutamine, and serine are examples of residues that engage in this type of interaction. Lastly, the ninth term represents the unfolding energy (Reference) for each amino acid. The total Prime Stability score outperforms all the individual components of the Prime energy function (Fig 3). Showing that the sum of all terms is a superior predictor of functionally disrupting mutations than any individual term.

### Dividing the dataset

To better understand the limitations and strengths of the five methods presented in Fig 1, we attempted to divide the dataset using three approaches. The first approach divides the data set on the basis of whether a mutation conserves or changes the net charge on the protein. The second approach divides the data on the basis of whether a mutation substantially changes the volume occupied by the residue (big->small or small->big). We classify a volume change “significant” if it changes the volume of the sidechain by > = the volume of an alanine->valine mutation. And lastly, we separate mutations that occur on the surface of the protein from mutations that are buried in the protein, using the relative accessible surface area of the wildtype side chain to determine if it is solvent exposed [23].

Mutations involving residues that are typically charged at neutral pH (D, E, R, K,) can be challenging for several reasons. For instance, depending on the protein environment, charged residues can occupy different protonation states, which can significantly alter their physio-chemical properties. Additionally, positively charged residues, such as lysine and arginine, contain a larger number of rotatable bonds relative to other amino acids, making prediction of the side chain conformation for these positively charged residues challenging. Due to these same types of challenges, other physics-based algorithms have also been documented to struggle with predicting changes in stability for mutations that change the net charge of a protein [24]. Thus, we evaluated whether the performance of Prime would improve upon removal of mutations that change the net charge of the protein.

**Fig 4:**
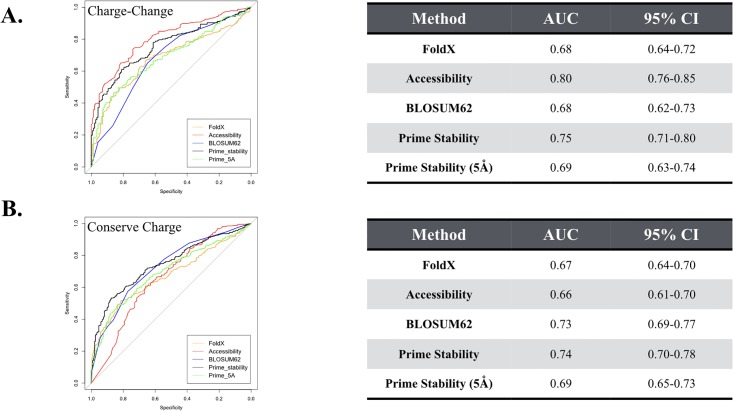
ROC curves separated by mutations that change in charge from mutations that conserve charge. (A) The charge-change dataset consisted of 369 mutations (B) The conserve-charge dataset consists of 623 mutations that conserve the net charge of the protein. The tables on the right list the area under the curve (AUC) values along with 95% confidence interval (CI) for the AUC values.

As seen in Fig 4A, Prime obtains an AUC of 0.75 on mutations changing the net charge of the protein. Surprisingly, this performance is on par with Prime’s performance on the entire dataset (AUC = 0.75) and very similar to the performance on the dataset involving mutations conserving the net charge of the protein (AUC = 0.74, Fig 4B). Only the accessibility metric showed substantial sensitivity to dividing the dataset in this way, obtaining an AUC of 0.66 on the entire conserve-charge set vs an AUC of 0.80 on mutations thought to change the net charge of the protein.

**Fig 5:**
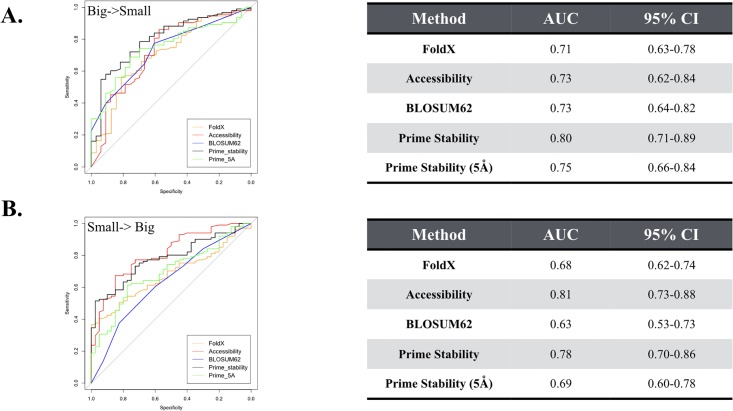
ROC curves separated by mutations that decrease in volume from mutations that increase in volume. (A) The big->small dataset consists of 126 mutations that decrease the net volume of the side chain (see Methods). (B) The small->big dataset consists of 141 mutations that increase the volume of the mutated side chain (see Methods). The tables on the right list the area under the curve (AUC) values along with corresponding 95% confidence interval (CI) for the AUCs.

We divided the dataset to separate big to small mutations from small to big mutations to determine if the added flexibility of the side chain relaxation protocol in Prime would benefit small to large mutations. Small to big mutations are defined as mutations whose side chain volume increases by more than the volume of an alanine to valine mutation (This was inversely used for big to small mutations). Interestingly, the 5 Å refinement protocol in Prime negatively impacted performance for the small to big mutations, reducing the AUC of Prime from 0.78 to 0.69 (Fig 5). Also, and perhaps not unexpectedly, the added flexibility did not improve Prime’s performance for the big to small mutations. This suggests that not perturbing the crystal structure is beneficial relative to allowing minor alterations of the side chains of the structure. This has previously been observed by Kellogg et al. when modifying the sampling protocol of the Rosetta energy function [25]. It is also worth noting that the BLOSUM62 substitution matrix struggled with the small to big mutations (AUC = 0.63) relative to the entire dataset (AUC = 0.71).

**Fig 6:**
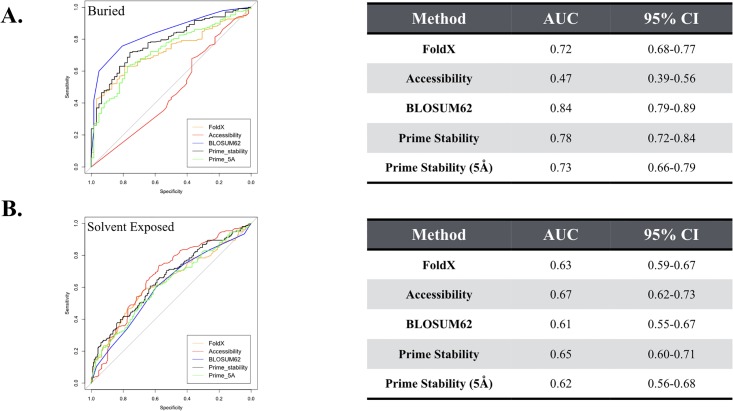
ROC curves separated by mutations that are buried in the protein from solvent exposed mutations. (A) The buried dataset consists of 292 mutations that are buried in the core of beta lactamase (< = 5% solvent exposed). (B) The solvent exposed dataset consists of 367 mutations that are solvent exposed (>20% solvent exposed). The tables on the right list the area under the curve (AUC) values along with corresponding 95% confidence interval (CI) for the AUCs.

It has been documented that positions at the surface of a protein are typically more tolerant to mutation than mutations at buried positions in a protein [26]. As a result, we were interested in whether the performance of the models tested in this study would be sensitive to whether a mutation appears at the surface of the protein or at a buried position (Fig 6). A position is defined as buried if the percent of solvent exposure of the wildtype amino acid is < = 5%. A position is defined as solvent exposed if the percent of solvent exposure of the wildtype residue is >20%. Predictably, the accessibility metric does not perform well at classifying functionally disrupting mutations at buried positions, getting an AUC value close to random (AUC = 0.47). In contrast, the BLOSUM62 matrix does exceptionally well at classifying mutations as functionally disrupting at buried positions, resulting in an AUC of 0.84. Prime’s performance on the buried set is very close to its performance on the entire dataset, resulting in an AUC of 0.78 vs. an AUC of 0.75 on the entire dataset. Added flexibility in Prime did not help Prime with buried mutations, resulting in an AUC of 0.73. Interestingly, all algorithms significantly struggle with classifying disrupting mutations on the surface of the protein. The AUCs range from 0.61 to 0.67.

## Conclusion

The MM-GBSA method in Prime is able to outperform all other methods tested in this study for predicting functionally disrupting mutations. And, perhaps more importantly, Prime works more robustly across various divisions of the dataset than the other methods tested in this study. The broad consistent performance of Prime likely reflects the fact that Prime is a physics-based approach. Excluding surface exposed mutations, the AUC values of Prime range from 0.74 to 0.80, which is a range of only 0.06. This is equal to the range of the 95% confidence interval on the entire dataset (Fig 2). However, the AUC values for the BLOSUM62 matrix range from 0.63 to 0.84. This is a variation of 0.21, a value that is more than three times larger than Prime’s variation. The range of AUC values obtained for the accessibility metric is even larger in magnitude, ranging from 0.47 (a nearly random model) to 0.81. The scoring function in FoldX, similar to Prime, uses physics-based terms. And thus, similar to Prime, FoldX also achieves a consistent performance, with AUC values ranging from 0.67 to 0.72. However, Prime is always able to outperform FoldX on all divisions of the dataset.

Beyond benchmarking Prime, we have attempted to improve Prime’s performance by introducing flexibility in the protein side chains within 5 Å of the residue being mutated. Intriguingly, this degrades performance across all divisions of the dataset, even for mutations that significantly increase the volume of the side chain being mutated. Others have made similar observations when validating scoring functions for protein stability. As observed in Kellogg et al., to properly introduce protein flexibility into the Rosetta energy function it is required to allow backbone atoms of the protein to move in conjunction with side chain motion [25]. Therefore, future studies using molecular-dynamics based methods such as free energy perturbation [27,28], which allow for complete protein flexibility, should also be benchmarked to determine how well these approaches can predict functionally disrupting mutations based on changes in protein stability.

**Fig 7:**
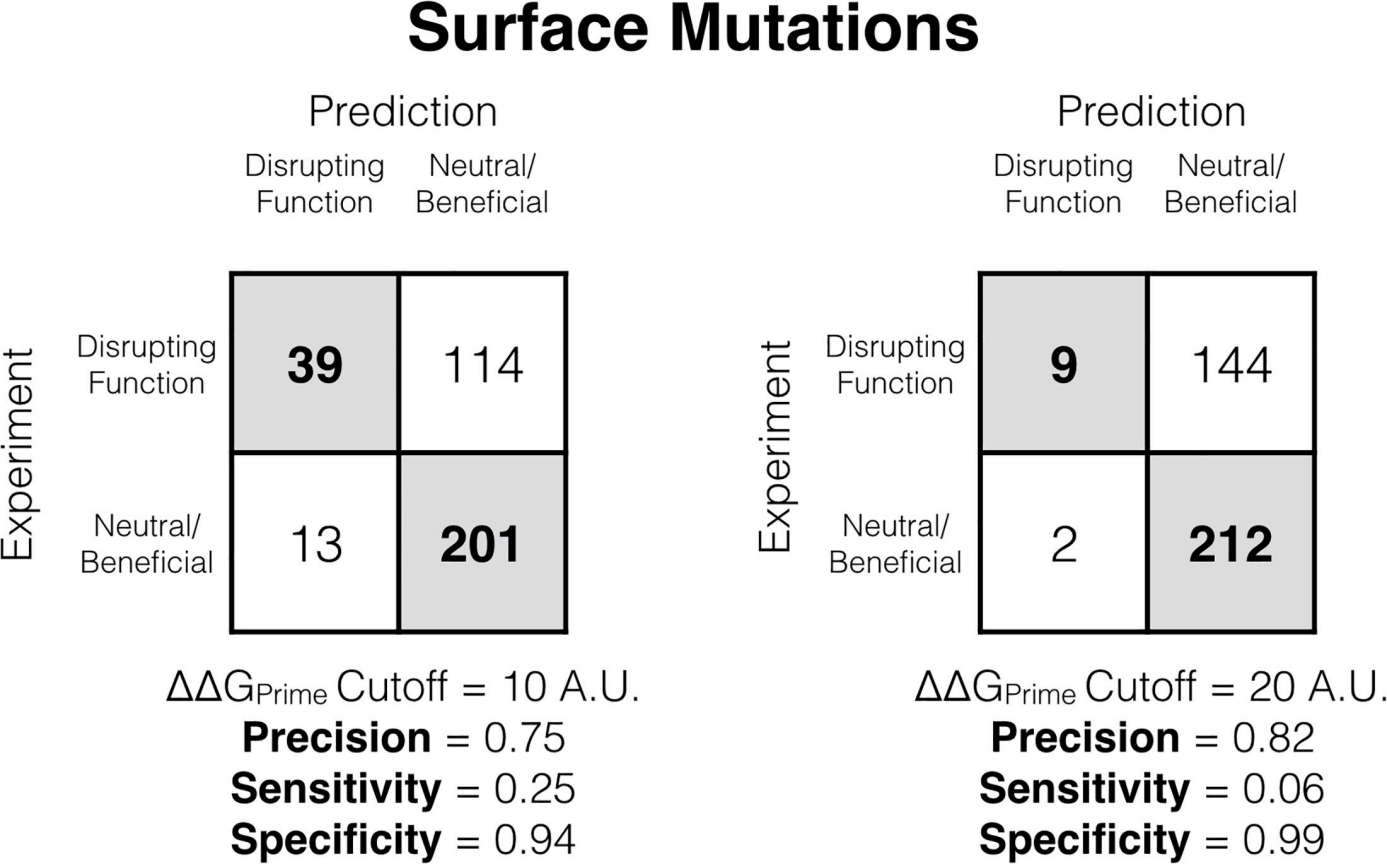
Two confusion matrices classifying functionally disrupting mutations on the surface using the Prime stability score. The left matrix uses a Prime energy cutoff of 10 prime energy units to classify functionally disrupting mutations from neutral/beneficial mutations. The matrix on the right uses a Prime energy cutoff of 20 prime energy units. The precision, sensitivity, and specificity are shown below the matrices.

When discussing the generality of Prime at predicting mutations that will disrupt function it is also paramount to discuss its shortcomings. Prime struggles on surface exposed positions, obtaining an AUC only of 0.65 (Fig 6). However, it is worth pointing out that when Prime predicts that a surface mutation does destabilize the protein (and thus disrupts function), the prediction is likely correct, as shown by the high precision and specificity scores in Fig 7 using two separate cutoff values for Prime. Prime’s limitation is that when it predicts that a mutation does not disrupt stability (and function) this may or may not be correct, as reflected by the low sensitivity values for two different Prime cutoffs.

**Fig 8:**
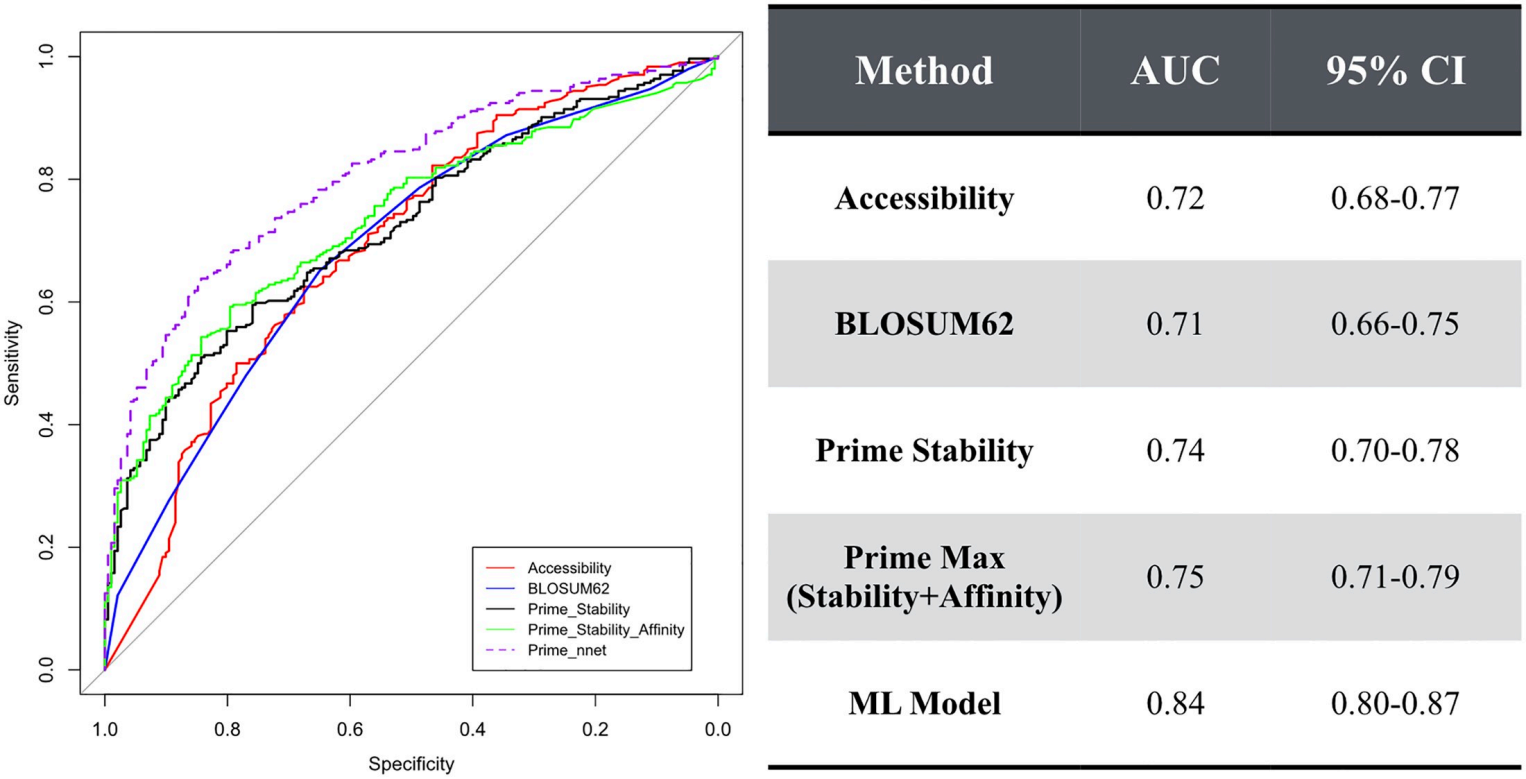
ROC curves using 495 mutations randomly sampled from the beta lactamase dataset. Prime Max refers to the maximum prime energy between the predicted change in stability and predicted change in affinity. The machine learning (ML) model refers to a single layer neural network trained on the other 495 mutations not included in this dataset. The area under the curve (AUC) values along with the corresponding 95% confidence intervals are shown in the table on the right.

The main challenge for mutations on the surface is that these mutations have several mechanisms for disrupting function. For instance, cysteine mutations on the surface may engage in disulfide bridges that prevent the protein from performing its function. And this may be why mutations to cysteine are 10 times more likely to result in functional disruption than to be neutral/beneficial. Additionally, mutations at the surface of a protein can interfere with crucial protein-protein interactions or protein-substrate interactions, which cannot be predicted from a crystal structure of just a protein monomer. However, in the case of beta-lactamase, we can use the crystal structure of beta lactamase in complex with an acylation transition state analog to approximate changes in binding affinity to the natural substrate. To do this while still accounting for changes in protein stability we took the maximum Prime score between the predicted change in stability and the predicted change in affinity for each mutation. The paradigm of this approach is that if a mutation is predicted to have a minor change in stability but is predicted to significantly destabilize substrate binding affinity the mutation would still be classified as functionally disrupting and vice versa. Unfortunately, this did not significantly improve the results, changing the AUC value from 0.74 to 0.75 (Fig 8). As a result, it appears that for this dataset, loss of affinity to substrate plays a nominal role.

Beyond the challenge of surface mutations, there are additional factors impeding the prediction of functionally disrupting mutations via protein stability predictions. For example, molecular chaperons have been shown to guide the folding of a protein whose unfolded state is actually lower in energy than its folded state [29]. This is done through kinetically trapping the protein in the folded state. And fascinatingly, these chaperones have been known to act heterogeneously on genetic variants of a protein causing the connection between protein stability and function to be further blurred [30].

Another significant limitation to applying Prime to predict functionally disrupting mutations is the ability to obtain an accurate structure of the protein target of interest. In this study, the 1BTL crystal structure provided a promising starting point to build a structure-based model of the beta-lactamase protein, only requiring hydrogen atoms to be added and optimized. However, despite the large size of the protein databank, the majority of proteins have not been crystalized [31]. Homology modeling provides a path to extend the protein databank for proteins closely related in sequence to those that have already been crystallized, and future studies should look into how well Prime will predict functionally disrupting mutations using homology models.

Finally, using a single-layer neural network, we integrated the best performing predictors on the entire beta lactamase dataset, which are accessibility, BLOSUM62, and Prime Max, into a single machine learning (ML) model. We trained the ML model by randomly sampling half the 990 single-point mutations, and testing on the remaining half. Interestingly, the model obtains an AUC of 0.84 (Fig 8). It is not clear if the ML model will generalize beyond the beta-lactamase dataset. However, it demonstrates the ability to unify the models under a single score. And such an approach may assist with integrating models that capture other mechanisms for disrupting function outside of protein stability—such as those described above. Lastly, despite the limitations described, accurate models of protein stability do capture a significant amount of the functionally disrupting mutations in the beta-lactamase dataset.

## Materials and methods

### Preparing 1BTL and 1AXB crystal structures

Schrodinger’s Protein Preparation Wizard (PrepWizard) was used to prepare all PDB structures for Prime [32]. The PrepWizard assigns bond orders, predicts protonation states, samples Asn/Gln/His flip states, removes select crystallographic waters, optimizes the H-bond network, and minimizes the structure. Default values were used for all parameters except the -propka_pH flag, which was set to pH 7.2. The following command line was used:

$SCHRODINGER/utilities/prepwizard -fillsidechains -propka_pH <pH> -NOJOBID <Input.pdb> <Output.mae>

### Running Residue Scanning

The Residue Scanning functionality in BioLuminate (18) version 18–3 was used to By default,For this work, the default sampling protocol was used via the following command line:

$SCHRODINGER/run $SCHRODINGER/mmshare- v32017/python/scripts/residue_scanning_backend.py jobname $jobname -fast—res_file <Resfile_name> -receptor_asl <Chain_name> -refine_mut prime_residue -dist 0.00 <Input_file.mae> -NJOBS 1 –NOJOBID

Due to limitations of the Residue Scanning application, mutations breaking covalent bonds in the structure were given a Prime score of 1000.0.

### Splitting the beta-lactamase dataset

As described, the beta-lactamase dataset has been divided using three approaches. The first approach separates the mutations that change the net charge of the protein from those that conserve the net charge of the protein. For generating the dataset of net-charge-changing mutations, mutations involving lysine, arginine, aspartic acid, and glutamic acid are used. If residues are mutated to or from these amino acids they are considered to change the net charge of the protein, unless the mutation remains positive (i.e. a lysine to an arginine or an arginine to a lysine) or remains negative (i.e. an aspartic acid to a glutamic acid or a glutamic acid to an aspartic acid). Mutations are considered to conserve the net charge of a protein if they do not involve lysine, arginine, aspartic acid, or a glutamic acid. With the exception being for mutations between lysine and arginine or between glutamic acid and aspartic acid.

To split the dataset by mutations that significantly increase (small->big) or decrease (big->small) in volume, we use van der Waals volumes reported by Darby and Creighton (1993) [33]. Changes in volume larger than an alanine to valine mutation (37 Å^3^) are considered to be a significant change in volume, and vice versa for mutations decreasing in volume. More stringent filters were considered, however, these resulted in unreliably small datasets.

Lastly, to classify mutations as buried or solvent exposed, the relative solvent accessible surface area is calculated for each position in the wildtype structure. This was done by dividing the solvent accessibility (Accessibility) metric by the maximum solvent accessible surface area of that amino acid in a tri-peptide according to Miller et al. (1987) [23]. Relative solvent accessible surface areas < = 0.05 were classified as buried. And relative solvent accessible surface areas >0.20 were considered solvent exposed. Glycine residues were excluded from this analysis.

### MIC score, FoldX, Accessibility, and BLOSUM62

The values for the MIC score, FoldX, Accessibility, and BLOSUM62 were obtained from Jacquier et al. [20].

### Calculating the area under the curve (AUC) and the machine learning (ML) model

R version 3.3.0 was used the calculate the receiver operating characteristic curves (ROC). R was used to calculate the area under the curve, the 95% confidence intervals train a neural network [34]. Specifically the pROC package and the nnet package in R were used [35,36].

## Supporting information

S1 Table Model scores for each mutation. (CSV)
